# Intraoperative management of brain-dead organ donors by anesthesiologists during an organ procurement procedure: results from a French survey

**DOI:** 10.1186/s12871-019-0766-y

**Published:** 2019-06-15

**Authors:** Benoit Champigneulle, Arthur Neuschwander, Régis Bronchard, Gersende Favé, Julien Josserand, Benjamin Lebas, Olivier Bastien, Romain Pirracchio, in collaboration with the SFAR research network

**Affiliations:** 1 Anesthesiology and Intensive Care Department, European Hospital Georges-Pompidou, Assistance Publique - Hôpitaux de Paris (AP-HP), 20 rue Leblanc, 75015 Paris, France; 2 West francilian network for organ and tissue procurement, Paris, France; 3 Agence de la Biomédecine, Direction Prélèvement Greffe Organes-Tissus, Saint-Denis La Plaine, France; 4 Paris Descartes University, Sorbonne Paris Cité, Paris, France; 5 Anesthesiology and Intensive Care Department, Hautepierre Hospital, Strasbourg, France; 6 Département de biostatistiques et d’informatique médicale, INSERM U-1153, Équipe ECSTRA, Université Paris Diderot; Hôpital Saint-Louis, AP-HP, Paris, France

**Keywords:** Anesthesia, Brain-dead donors, Organ procurement, Survey

## Abstract

**Background:**

This study aimed at describing usual anesthetic practices for brain-dead donors (BDD) during an organ procurement (OP) procedure and to assess the knowledge and self-confidence of French anesthesiologists with this practice.

**Methods:**

An electronic and anonymous survey with closed-questions about anesthetic management of BDD was distributed to French anesthesiologists via the mailing list of the French Society of Anesthesiology and Intensive Care Medicine.

**Results:**

Four hundred fifty-eight responses were analyzed. Respondents were mainly attending physicians with more than 10 years of clinical experience. 78% of them declared being cognizant of guidelines regarding management of BDD. Advanced hemodynamic monitoring and endocrine substitution were rarely considered by respondents (31 and 35% of respondents, respectively). 98% of the respondents used crystalloids for fluid resuscitation. During the procedure, use of neuromuscular blockers, opioids and sedative agents were considered by respectively 84, 61 and 27% of the respondents. A very high level of agreement (10 [8–10], on a ten-points Likert-style scale) was reported concerning the expected impact of intraoperative anesthetic management on the primary function of grafts.

**Conclusions:**

Declared anesthetic practice appeared in accordance with guidelines concerning organ donor management in the ICU. Further studies are needed to evaluate the specific impact of intraoperative management during this procedure and thus the need for specific anesthetic guidelines.

**Electronic supplementary material:**

The online version of this article (10.1186/s12871-019-0766-y) contains supplementary material, which is available to authorized users.

## Background

Brain-dead donors (BDD) currently remain the primary source of grafts for solid organ transplantation across the world [1, 2]. In this context, appropriate management of organ donors from the diagnosis of brain death to the end of the organ procurement (OP) procedure is of paramount importance to optimize the function of potential grafts. Intensive care (ICU) management of BDD is well-codified. This is an active research field [3] and many guidelines were published and regularly updated over the last years [4–7]. Conversely, intraoperative and anesthetic management of the donor during the OP procedure is far less codified [2, 8]. French guidelines mainly specify to follow the same organ resuscitation strategy initiated in the ICU and only specify that the use of neuromuscular blocking (NMB) agents and analgesics are justified [5]. Because of the lack of specific recommendations on the intraoperative and anesthetic management of OP procedure, we hypothesize that the practices are disparate.

In this context, we performed a French national survey on intraoperative management of BDD by anesthesiologists. The aims of the study were i) to describe intraoperative anesthetic practices for BDD during an OP procedure; and ii) to evaluate the knowledge of French anesthesiologists on this specific practice.

## Methods

### Questionnaire

We conducted a French national survey on intraoperative anesthetic management of BDD during the OP procedure. The questionnaire included 33 closed-questions and one open-ended question (see the Additional file 1). The questionnaire was anonymous and subdivided into seven sections taking into account the domains covered by the guidelines [4–7]: general and demographic data, per-operative monitoring, hemodynamic management, metabolic management, respiratory management, anesthetic drugs employment and personal feeling about the procedure. Intraoperative use of donor management goals (DMGs) was also evaluated. For questions on standard practice, a five-point Likert-style scale was provided (ranging from *never* to *always*); for questions covering provider’s perception of the OP procedure, a ten-point Likert-style scale ranging from 1 (*certainly not agree*) to 10 (*absolutely agree*) was used. Before broadcasting, the questionnaire was tested and approved by the anesthesiologists of our tertiary teaching hospital (European Georges Pompidou hospital, AP-HP, Paris, France).

According to the French law, no ethic committee approval was required for this anonymous survey intended to health professionals.

### Survey processing

The questionnaire was meant to target all French anesthesiologists. Expected time to complete the questionnaire was less than 10 min. An electronic form of the survey was compiled using SurveyMonkey® (https://www.surveymonkey.com). In early September 2017, French anesthesiologists were invited to answer the survey via an email, sent through the mailing-list of the French Society of Anesthesia and Intensive Care (*Société Française d’Anesthésie-Réanimation*, SFAR). The survey was available *on-line* during a 4-month period (until end of December 2017). A follow-up email was sent 2 months after the first e-mail, in November 2017.

### French local organization for organ procurement

In France, OP from BDD is only performed in hospitals accredited by the French Biomedicine Agency which is the only state agency that regulates and organizes OP and transplantation in France. In 2017, 182 medical centers were accredited in France for OP from BDD. In each center, a dedicated local team (including specialized nurses) is involved from the identification of the potential BDD in the ICU to the admission of the deceased person in the mortuary room. Local OP teams are not directly involved in the medical management of the BDD in the ICU or in the OR which remains under the purview of the attending physician. National French guidelines regarding BDD management were updated in 2005 by the French society of anesthesia and intensive care medicine and the Biomedicine Agency [5]. Authorized centers are incited to develop local procedures based on guidelines.

### Data analysis

Results are reported as count (%) for categorical variables and median (25th–75th percentiles) for continuous variables. For questions pertaining to practice, in order to simplify the interpretation of the results, the answers “never” and “seldom” were grouped, as well as the answers “always”, “often” and “regularly”. Reported responses were compared between more junior respondents (including residents and doctors with less than 10 years of clinical experience) and more senior respondents (doctors with more than 10 years of clinical experience). The Pearson chi-square test was used to compare categorial variables. Analyses were performed using Microsoft® Excel software (2017) and SPSS software version 20 (SPSS, Chicago, IL, USA). All tests were 2-sided with *p* < 0.05 considered to define statistical significance.

## Results

### Respondent characteristics

Four hundred and fifty-eight anesthesiologists answered the survey during the study period. General characteristics of the respondents are described in Table 1. Among the respondents, 359 (78%) declared having knowledge on ICU BDD management guidelines; less junior respondents claimed being cognizant of these recommendations than senior respondents: 72% vs. 86% (*p* < 0.001) (Additional file 1: Table S1).

**Table 1:** Characteristics of the respondents

	Respondents (*n* = 458)
Type of institution	
University hospital	267 (58%)
Non-university hospital	191 (42%)
Function of the respondent	
Resident	48 (10%)
Fellow	53 (12%)
Attending physician	335 (73%)
Professor	22 (5%)
Field of activity^a^	
Full-time anaesthesiology	188 (46%)
Full-time ICU	42 (10%)
Shared activity (both anaesthesiology and ICU)	180 (44%)
Professional experience > 10 years^a^	209 (51%)
Numbers of OP procedures occurred last year per establishment	
< 5 procedures	55 (12%)
5–10 procedures	95 (21%)
10–20 procedures	132 (29%)
> 20 procedures	176 (38%)
Numbers of BDD personally managed in the OR by respondent during last year	
None	71 (16%)
1–4	307 (67%)
≥ 5	80 (17%)
Existence of a written protocol for anaesthetic management of BDD	197 (76%)

Data are expressed as *n* (%)

^a^Excluding residents

*ICU* intensive care unit, *BDD* brain dead donor, *OP* organ procurement, *OR* operating room

### Per-operative monitoring

**Fig. 1:**
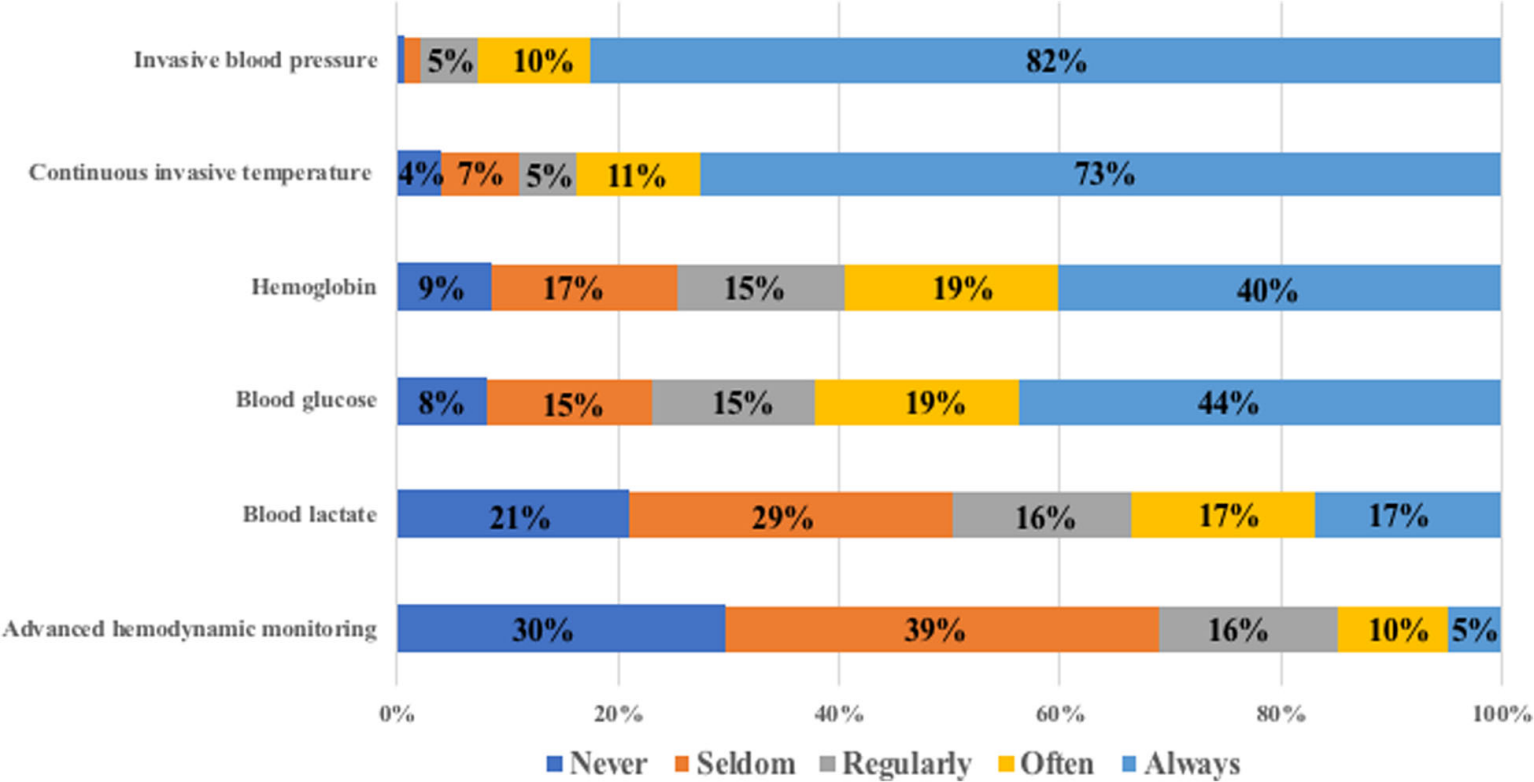
Detailed answers concerning the use of complementary monitoring during the organ procurement procedure

Almost all respondents (98%) declared always using the standard intraoperative monitoring equipment required by the SFAR (i.e., electrocardiogram, blood pressure, SpO_2_ and end-tidal CO_2_) during the OP procedure. Detailed answers concerning other optional monitoring are presented in the Fig. 1. Invasive blood pressure and invasive temperature monitoring were reported to be frequent (97 and 89%, respectively). Blood lactate or hemoglobin monitoring during the procedure is less frequent (50 and 74%, respectively). Most of the respondents (69%) did not report the use of advanced hemodynamic monitoring in this context. When an advanced hemodynamic monitoring is used, pulse pressure analysis seems to be the most commonly used device in this setting (63%).

### Donor management during organ procurement

*DMGs*: 358 (78%) respondents reported to use pre-specified DMGs during the OP procedure, without significant difference according to the level of seniority (Additional file 1: Table S1). This included: targeted mean arterial pressure range of 60–70 mmHg for 305 (67%) respondents and temperature > 35 °C for 96% of the respondents.

*Hemodynamic management* (Additional file 1: Figure S1) presents the distribution of the answers concerning intraoperative fluid management. Briefly, 95% of the responding anesthesiologists did not used starches for fluid resuscitation and 98% of them reported using crystalloids in this context. The most frequently used crystalloids were ringer lactate (45%) and 0.9% saline (38%). A hemoglobin threshold of 7 g.dL^− 1^ was considered for transfusion by 298 (65%) respondents whereas 21 (5%) of them considered that blood transfusion was not indicated in this setting.

*Pulmonary management*: most anesthesiologists (93%) reported implementing a protective ventilation strategy in the OR and 92% of them declared realizing recruitment maneuvers during the procedure (routinely in 55% of cases). More junior respondents appeared to implement more often a protective ventilation than the senior respondents (Additional file 1: Table S1).

*Endocrine substitution*: during the OP procedure, hormonal resuscitation was not considered by 65% of the respondents, without significant difference between respondents (Additional file 1: Table S1). When hormonal replacement therapy was considered, hormone substitution protocol varied substantially across practitioners except for triiodothyronine that was not used by 93% of the respondents (Additional file 1: Figure S2).

### Anesthetic management during organ procurement

**Fig. 2:**
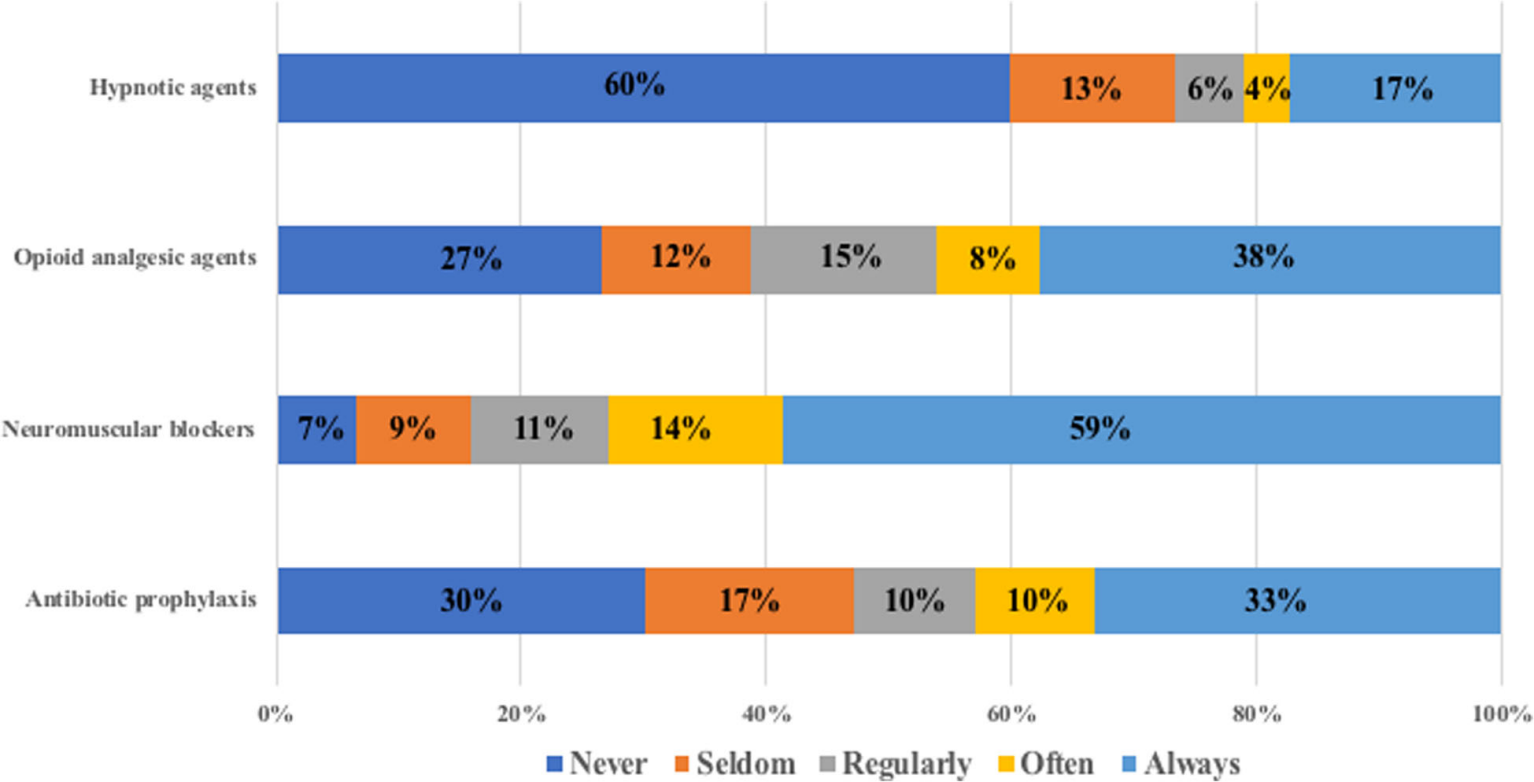
Declared practices concerning anesthetic drugs utilization during the organ procurement procedure

Responses concerning anesthetic drugs utilization are reported in the Fig. 2. Use of NMB, opioids and sedative agents was considered by respectively 84, 61 and 27% of the respondents. Among anesthesiologists who declared using a sedative agent for the procedure, the most popular agents were volatile anesthetics (65%). For 76% of the respondents, administration of unfractionned heparin is only done when asked by surgeons or the OP coordinator.

### Provider’s perceptions

Provider’s perception concerning the anesthetic management of BDD was evaluated using a numeric scale ranging from 1 to 10. The presence of an attending anesthesiologist in the OR during the procedure was deemed necessary with an agreement of 8 (7–10). The question of feeling enough prepared for OP procedure was rated with a score of 6 (5–8). Consistently, responding anesthesiologists considered that further specific recommendations on anesthetic management of BDD would be useful (score of agreement: 9 (8–10)). A very high level of agreement (10 (8–10)) was also reported concerning the expected impact of intraoperative anesthetic management on the primary function of grafts.

## Discussion

Our study aimed at describing current practices and perceptions of a large panel of anesthesiologists on the intraoperative management of BDD. The few existing recommendations on the subject are essentially based on expert opinions or extrapolated from ICU guidelines for BDD management [1, 2]. Briefly, our survey suggests that anesthetic practices concerning monitoring, DMGs, fluid resuscitation and ventilatory management are in agreement with the current ICU guidelines despite significant difference between the respondents according to their level of seniority [4–7]. Particularly, more senior anesthesiologists were more likely to report knowledge of the current ICU guidelines. Conversely, more junior doctors appeared more likely to use a protective ventilation strategy, maybe because younger anesthesiologists were more trained in the era of lung-protective ventilation [9].

In the absence of specific anesthetic guidelines, maintaining the same level of care implemented in ICU is fundamental, as raised in a recent review [2]. Recent findings concerning the use of mild hypothermia (34–35 °C) to improve renal grafts recovery does not seem to be integrated into current practices [3]. The low reported use of hormone substitution may be surprising. However, this is in agreement with the French guidelines which do not support systematic endocrine substitution (except for vasopressin analogs in case of diabetes insipidus) [5]. This point differs with north-American guidelines [6, 7]. However, the level of evidence concerning the benefit of hormonal substitution (such as corticosteroids supplementation) remains relatively limited in this context [10]. The use of an advanced hemodynamic monitoring during the OP procedure did not seem to be a part of standard practices for a majority of the respondents in our survey. To our knowledge, there is no published data concerning the best device for hemodynamic assessment in this context. If pulse pressure variations could be use in the setting of brain death to guide fluid therapy [11], a recent randomized study failed to demonstrate any benefit (in terms of number of organs transplanted) of using a protocolized fluid therapy based on pulse-pressure variation and cardiac index [12].

The use of anesthetic drugs during the OP procedure remains a matter of debate [13]. In the context of brain death, the goal of anesthetic medications is essentially to control any possible hemodynamic and/or motor response resulting from spinal cord reflexes, thereby justifying the use of neuromuscular blocking and analgesic agents during the OP procedure [5]. Opioids alone may be insufficient to control catecholamine release induced by surgical stimulation [14]. The use of volatile anesthetic, as “vasodilator agents”, may thus be justified [2, 8]. In addition, a potential beneficial effect of volatile anesthetic agents on ischemia-reperfusion injuries has been suggested and could further justify their use in this context [2, 4, 8]. However, the level of scientific evidence remains relatively limited and further investigations are needed.

Finally, the most interesting finding of our study may be the high perceived impact of intraoperative management on the primary function of the grafts. Consistently the responders reported having high expectations from the scientific societies to produce specific guidelines on anesthetic management for BDD. Further research in the area is needed to give consistency to future evidence-based guidelines.

This declarative study carries some limitations. The survey was sent to an unselected panel of French anesthesiologists, members of the French Society of Anesthesia and Intensive Care Medicine. Although this panel is supposed to be representative of the overall population of practicing anesthesiologists, we cannot rule out some degree of responder bias and thus the results may not be fully representative of the actual current practices. Furthermore, our results might not reflect the practices outside France, especially since there are some known discrepancies between current national guidelines concerning BDD management [5–7].

## Conclusions

Declared anesthetic practices concerning intraoperative management of BDD during organ procurement procedures are in accordance with national French guidelines on organ donor management. Further studies are needed to investigate this specific area of donor management, to evaluate the potential impact of specific interventions (such as the use of anesthetic agents, hormone substitution or meeting intraoperative specific donor management goals…) on the graft function after transplantation and to build future high-quality anesthetic guidelines on intraoperative management of BDD.

## Additional file

Additional file 1: Survey form and additionnal figures and table. (DOCX 93 kb)

## Data Availability

The datasets used and/or analysed during the current study are available from the corresponding author on reasonable request.

## Abbreviations

AP-HP: Assistance Publique - Hôpitaux de Paris
BDD: Brain-dead donors
DMGs: Donor management goals
ICU: Intensive Care Unit
NMB: Neuromuscular blocking
OP: Organ procurement
OR: Operating room
SFAR: French Society of Anesthesia and Intensive Care (*Société Française d’Anesthésie-Réanimation*)
