# Supplementary material for: Intraoperative management of brain-dead organ donors by anesthesiologists during an organ procurement procedure: results from a French survey
Source: BMC Anesthesiol. 2019 Jun 15;19:108. doi: 10.1186/s12871-019-0766-y (PMC6570868; doi:10.1186/s12871-019-0766-y)
Supplement: Supplementary file 1 — Survey form and additionnal figures and table. (DOCX 93 kb) [file 12871_2019_766_MOESM1_ESM.docx]

**Additional file 1**

**Intraoperative management of brain-dead organ donors by anaesthesiologists during an organ procurement procedure: Results from a French survey**

Benoit Champigneulle, MD^1,2*^; Arthur Neuschwander, MD^1^; Régis Bronchard, MD^3^; Gersende Favé, MD^1,4^; Julien Josserand, MD^1^; Benjamin Lebas, MD^5^; Olivier Bastien, MD-PD^3^; Romain Pirracchio, MD-PhD^1,4,6^ in collaboration with the *SFAR research network*.

1. Anaesthesiology and Intensive Care Department, European Hospital Georges-Pompidou, AP-HP, Paris, France.

2. West francilian network for organ and tissue procurement, Paris, France.

3. Agence de la Biomédecine, Direction Prélèvement Greffe Organes-Tissus, Saint-Denis La Plaine, France.

4. Paris Descartes University, Sorbonne Paris Cité, Paris, France.

5. Anaesthesiology and Intensive Care Department, Hautepierre Hospital, Strasbourg, France

6. Département de biostatistiques et d’informatique médicale, INSERM U-1153, Équipe ECSTRA, Université Paris Diderot; Hôpital Saint-Louis, AP-HP, Paris.

***Corresponding author:** Pr Romain Pirracchio, Anesthesiology and Intensive Care Department**,** Georges Pompidou European Hospital, Assistance Publique - Hôpitaux de Paris (AP-HP)**,** 20 rue Leblanc, 75015 Paris, France.

Phone: +33 156 092 714; Fax: +33 156 092 551

E-mail: [romain.pirracchio@aphp.fr](mailto:romain.pirracchio@aphp.fr)

**Electronic Survey form**

***General and demographic data***

1. In which type of hospital are you working?

☐ Non-University Hospital ☐ University Hospital

2. What is your medicine grade?

☐ Resident ☐ Fellow ☐ Attending physician ☐ Professor

3. For residents, in which training semester are you?

☐ 1 ☐ 2 ☐ 3 ☐ 4 ☐ 5 ☐ 6 ☐ 7 ☐ 8 ☐ 9 ☐ 10

4. For others medicine grade, how long have you been practicing?

☐ < 5 years ☐ 5-10 years ☐ 10-20 years ☐ >20 years

5. What is your professional occupation?

☐ Only anesthesia ☐ Only intensive care ☐ Both anesthesia and intensive care

6. How many organ procurement procedures were (approximately) realized at your institution last year?

☐ < 5 ☐ 5-10 ☐ 10-20 ☐ >20

7. How many BBD have you personally taken care of in the operating room over the past year?

☐ 0 ☐ 1 ☐ 2 ☐ 3 ☐ 4 ☐ ≥ 5

8. Does your institution have a written protocol for anesthetic management of organ procurement procedures?

☐ No ☐ Yes

9. Do you use pre-specified donors management goals during the organ procurement procedure?

☐ No ☐ Yes

10. Are you aware of recommendations about medical management of BBD in the ICU published in 2005 on the behalf of the French Society of Anesthesiology and Intensive Care and the Biomedicine Agency?

☐ No ☐ Yes

***Per-operative monitoring***

11. Which type of monitoring do you use during organ procurement procedure on BDD?

- Minimum essential monitoring (i.e.: pulse oximeter, non-invasive blood pressure, ECG, capnography):

☐ Never ☐ Seldom ☐ Regularly ☐ Often ☐ Always

- Continuous invasive temperature monitoring (i.e. urinary bladder or esophageal probe):

☐ Never ☐ Seldom ☐ Regularly ☐ Often ☐ Always

- Blood glucose monitoring (at least one measure by procedure):

☐ Never ☐ Seldom ☐ Regularly ☐ Often ☐ Always

- Hemoglobin monitoring (capillary or venous blood simple, at least one measure by procedure:

☐ Never ☐ Seldom ☐ Regularly ☐ Often ☐ Always

- Urine output monitoring (at least one measure by procedure):

☐ Never ☐ Seldom ☐ Regularly ☐ Often ☐ Always

- Invasive blood pressure monitoring:

☐ Never ☐ Seldom ☐ Regularly ☐ Often ☐ Always

- Blood lactate monitoring:

☐ Never ☐ Seldom ☐ Regularly ☐ Often ☐ Always

- Advanced hemodynamic monitoring:

☐ Never ☐ Seldom ☐ Regularly ☐ Often ☐ Always

12. If you happen to use advanced hemodynamic monitoring, which type of device do you mostly used?

☐ Pulse pressure analysis device ☐ Esophageal Doppler

☐ Transesophageal echocardiography ☐ Pulmonary artery catheterization

***Hemodynamic management***

13. Which type of fluid do you use for fluid resuscitation during organ procurement procedure?

- Crystalloids:

☐ Never ☐ Seldom ☐ Regularly ☐ Often ☐ Always

- Synthetic colloids:
  - Gelatin

☐ Never ☐ Seldom ☐ Regularly ☐ Often ☐ Always

- - Starches

☐ Never ☐ Seldom ☐ Regularly ☐ Often ☐ Always

- Albumin:

☐ Never ☐ Seldom ☐ Regularly ☐ Often ☐ Always

14. Which type of crystalloid is the more frequently used at your institution?

☐ Ringer Lactate ☐ Saline ☐ Plasmalyte ☐ Other

15. If you monitor hemoglobin during an organ procurement procedure, which transfusion threshold does you use?

☐ Restrictive threshold (i.e. 7 g/dL) ☐ Liberal threshold (i.e. 10 g/dL)

☐ Other threshold ☐ Not hemoglobin monitoring

☐ Transfusion is not indicated in BDD

16. What is your mean arterial pressure target during an organ procurement procedure in a BDD?

☐ No specific target ☐ 50-60 mmHg ☐ 60-70 mmHg ☐ > 70 mmHg

17. If you use an advanced hemodynamic monitoring, are you focusing on optimizing any of the following parameter during an organ procurement procedure in a BDD?

☐ cardiac output (or stroke volume)

☐ venous oxygen saturation

☐ pulse pressure variation (or stroke volume variation)

☐ pulmonary artery pressure

☐ blood lactate concentration

☐ not using any advanced monitoring

***Metabolic management***

18. If you use monitoring temperature during organ procurement procedures, what is your body temperature target?

☐ 34-35°C ☐ 35-36°C ☐ 36-37°C ☐ no temperature monitoring

19. If you monitor urine output during organ procurement procedures, what is your output target?

☐ < 1 mL.kg^-1^.h^-1^ ☐ 1 - 1,5 mL.kg^-1^.h^-1^

☐ > 2 mL.kg^-1^.h^-1^ ☐ no urine output monitoring

20. During an organ procurement procedure in a BDD, do you administer hormone replacement therapy?

☐ Never ☐ Seldom ☐ Regularly ☐ Often ☐ Always

21. If you happen to administer hormone replacement therapy, which hormone do you administer?

- Corticosteroids:

☐ Never ☐ Seldom ☐ Regularly ☐ Often ☐ Always

- Insulin:

☐ Never ☐ Seldom ☐ Regularly ☐ Often ☐ Always

- Tri-iodothyronine (T3):

☐ Never ☐ Seldom ☐ Regularly ☐ Often ☐ Always

- Vasopressin or DDAVP:

☐ Never ☐ Seldom ☐ Regularly ☐ Often ☐ Always

***Drugs management***

22. During an organ procurement procedure in a BDD, do you use any sedative agent?

☐ Never ☐ Seldom ☐ Regularly ☐ Often ☐ Always

23. If you happen to use sedative agents, which type of drug do you mostly used?

☐ Propofol ☐ Inhaled anesthetic agent ☐ Other

24. During an organ procurement procedure in a BDD, do you use opioids?

☐ Never ☐ Seldom ☐ Regularly ☐ Often ☐ Always

25. During an organ procurement procedure in a BDD, do you use neuromuscular blockers?

☐ Never ☐ Seldom ☐ Regularly ☐ Often ☐ Always

26. During an organ procurement procedure in a BDD, in absence of antibiotic treatment indicated for a proven infection in the donor, do you use any antibiotic prophylaxis?

☐ Never ☐ Seldom ☐ Regularly ☐ Often ☐ Always

27. During an organ procurement procedure in a BDD, do you use unfractionated heparin?

☐ Never

☐ Systematically just before vascular clamping

☐ Systematically at any time of the procedure

☐ Only if asked by the surgical team or the organ procurement coordinator

***Respiratory management***

28. During an organ procurement procedure, do you apply a protective ventilation strategy (i.e. tidal volume of 6-8 mL/kg with 8-10 cmH20 PEEP)?

☐ Never ☐ Seldom ☐ Regularly ☐ Often ☐ Always

29. During an organ procurement procedure, do you realize pulmonary recruitment maneuvers?

☐ Never

☐ Only in case of oxygen desaturation

☐ Only after disconnection from the ventilator

☐ Only if asked by the surgical team or the organ procurement coordinator

☐ Systematically in case of lung procurement

***Personal feeling about anesthesia and organ procurement procedure***

30. On a scale of 1 (certainly not agree) to 10 (absolutely agree), do you think that a graduate anesthetist must be present in the operating room during the organ procurement procedure in a BDD?

☐ 1 ☐ 2 ☐ 3 ☐ 4 ☐ 5 ☐ 6 ☐ 7 ☐ 8 ☐ 9 ☐ 10

31. On a scale of 1 (certainly not agree) to 10 (absolutely agree), do you think to be enough about the anesthetic management of BDD?

☐ 1 ☐ 2 ☐ 3 ☐ 4 ☐ 5 ☐ 6 ☐ 7 ☐ 8 ☐ 9 ☐ 10

32. On a scale of 1 (certainly not agree) to 10 (absolutely agree), do you think that further expert’s recommendations could change your practice?

☐ 1 ☐ 2 ☐ 3 ☐ 4 ☐ 5 ☐ 6 ☐ 7 ☐ 8 ☐ 9 ☐ 10

33. On a scale of 1 (certainly not agree) to 10 (absolutely agree), do you think that anesthetic management of BDD could influence further graft primary function?

☐ 1 ☐ 2 ☐ 3 ☐ 4 ☐ 5 ☐ 6 ☐ 7 ☐ 8 ☐ 9 ☐ 10

34. Do you have any specific comment about the anesthetic management of the brain-dead organ donors?

………………..………………..………………..………………..………………..…………….

**Figure S1:** Reported practices concerning fluid resuscitation.****

**Figure S2:** Reported practices concerning hormonal supplementation.

**Table S1:** Analysis of pertinent responses to the survey according to the experience of the respondents.

| Variable | Junior  respondents ^a^  (*n*=249) | Senior  respondents ^b^  (*n*=209) | *p* value |
| --- | --- | --- | --- |
| Declared knowledge of national guidelines concerning BDD management  Yes  No | 179 (72%)  70 (28%) | 180 (86%)  29 (14%) | <0.001 |
| Declared use of pre-specified DMG during the OP procedure  Yes  No | 194 (78%)  55 (22%) | 164 (78%)  45 (22%) | 0.89 |
| Reported use of advanced hemodynamic monitoring ^c^  Never  Seldom  Regularly  Often  Always | 75 (30%)  108 (44%)  37 (15%)  19 (8%)  8 (3%) | 59 (29%)  69 (34%)  36 (17%)  26 (13%)  14 (7%) | 0.06 |
| Reported implantation of a protective ventilation in OR  Never  Seldom  Regularly  Often  Always | 4 (2%)  11 (4%)  15 (6%)  49 (20%)  170 (68%) | 3 (1%)  12 (6%)  46 (22%)  45 (22%)  103 (49%) | <0.001 |
| Reported administration of hormonal replacement therapy  Never  Seldom  Regularly  Often  Always | 81 (32%)  76 (31%)  41 (16%)  29 (12%)  22 (9%) | 63 (30%)  76 (36%)  36 (17%)  24 (12%)  10 (5%) | 0.40 |
| Reported administration of antibiotic prophylaxis during the OP procedure  Never  Seldom  Regularly  Often  Always | 85 (34%)  43 (17%)  19 (8%)  24 (10%)  78 (31%) | 53 (25%)  35 (17%)  26 (12%)  21 (10%)  74 (36%) | 0.19 |
| Reported use of NMB agents during the OP procedure  Never  Seldom  Regularly  Often  Always | 17 (7%)  19 (8%)  28 (11%)  39 (15%)  146 (59%) | 13 (7%)  24 (11%)  23 (11%)  26 (12%)  123 (59%) | 0.61 |
| Reported use of opioids during the OP procedure  Never  Seldom  Regularly  Often  Always | 63 (25%)  32 (13%)  27 (11%)  23 (9%)  104 (42%) | 59 (28%)  23 (11%)  43 (21%)  15 (7%)  69 (33%) | 0.03 |
| Reported use of sedative agents during the OP procedure  Never  Seldom  Regularly  Often  Always | 143 (58%)  35 (14%)  13 (5%)  10 (4%)  48 (19%) | 131 (63%)  27 (13%)  13 (6%)  7 (3%)  31 (15%) | 0.69 |
| Data are expressed as *n* (%).  BDD, brain-dead donors; DMG, donor management goals; OP, organ procurement; OR, operating room; NMB, neuromuscular blocking.  ^a^ Including residents and doctors with a professional experience < 10 years.  ^b^ Including doctors with a professional experience > 10 years.  ^c^ Data missing for seven responders (1.5%). | | | |
